# Physicochemical Characterization of *Detarium microcarpum* Seeds from Northern Benin

**DOI:** 10.1155/2022/7722138

**Published:** 2022-07-28

**Authors:** Adamou Gani Issa, Andriano Jospin Djossou, Mouaïmine Mazou, Guy Alain Alitonou, Fidèle Paul Tchobo

**Affiliations:** Training and Research Laboratory in Applied Chemistry, Polytechnic School of Abomey-Calavi, University of Abomey-Calavi, Benin (LERCA/EPAC/UAC-Bénin), 01 BP 2009 Cotonou, Benin

## Abstract

The objective of this work is to determine the physicochemical characteristics of the *Detarium microcarpum* seeds from North Benin, specially the proximate composition, colour, minerals, and antinutritional factors using standard analytical methods. The results show that the contents of moisture, protein, total sugars, lipid, crude fiber, and ash ranged, respectively, from 10.85 to 14.69%, from 13.54 to 17.82%, from 19.69 to 32.04%, from 8.68 to 11.90%, from 19.78 to 34.24%, and from 1.5 to 3.49%. The luminance (l∗), red saturation (a∗), and yellow saturation (b∗) have, respectively, ranged from 60.45 to 67.64, from 5.44 to 8.86, and from 8.24 to 9.28. Seeds contain interesting contents of potassium, calcium, magnesium, manganese, sodium, and iron; they have, respectively, varied from 6141.88 to 12305.16 mg/kg, from 1254.47 to 2168.62 mg/kg, from 1298.87 to 2533.06 mg/kg, from 75.18 to 307.23 mg/kg, from 53.52 to 136.19 mg/kg, and from 28.46 to 181.42 mg/kg. The investigation of antinutritional factors indicates the presence of oxalates, phytates, total phenolic compounds, and saponins with contents that have varied, respectively, from 1.01 to 2.36%, from 0.37 to 0.87%, from 3.13 to 7.61%, and from 1.35 to 4.59%. On average, the physicochemical characteristics of the Sudanian and Sudano-Guinean zones are similar, except for total sugar content.

## 1. Introduction

In the world, there is an urgent need for new food plants or new sources to meet the nutritional needs of the ever-growing populations. Indeed, according to the Food and Agriculture Organization of the United Nations (FAO), the prevalence rate of undernourishment is 9.9% in the world, 21% in Africa [1], and 7.4% in Benin [2].

Food insecurity persists in most African countries due to the increase in demographic pressure, the insufficiency of basic cereals [3]. The consequences of this situation are mainly hunger, malnutrition, and the increase of food-related diseases despite the availability of certain plant resources [4, 5]. Therefore, utilizing plant resources that produce edible fruits that are not exploited or not used optimally and available locally for food processing in developing countries can be an approach to achieve food security [6]. Indeed, nontimber forest products (NTFPs) for food provide food security, which contribute to household economic development [7, 8]. These are wild or cultivated plants that provide economic and food survival support for local populations [9]. According to the World Health Organization (WHO), wild plants are involved in meeting the health and dietary needs of 80% of people living in developing countries [10]. *Detarium microcarpum Guill. & Perr.* (Fabaceae-Caesalpinioideae) is one of these plant resources, present in arid regions of West and Central Africa from Senegal and Gambia to Sudan [11], whose fruits are underexploited. The sweet pulp of the fruit is consumed raw at maturity while the seeds are almost unexploited [12, 13]. However, the cooked seeds are sometimes used to thicken soup for human consumption [13]. Observations by [14] in Mali show that farmers sell the fruit at a loss, because they are unaware that the seed can also bring in money. The use of local edible materials requires being informed on the physicochemical characteristics of these materials [15], because their better exploitation in the nutritional field depends on it [6].

Physicochemical characterization has been reported on *Detarium microcarpum* seeds collected in Nigeria [16–21]. However, no studies seem to have been conducted on the physicochemical characteristics in relation to the provenance of *Detarium microcarpum* seeds. The objective of the present work is to determine the physicochemical characteristics of *Detarium microcarpum* seeds from North Benin and to study the variation of these characteristics according to the origin of the seeds.

## 2. Materials and Methods

### 2.1. Plant Material

**Figure 1:**
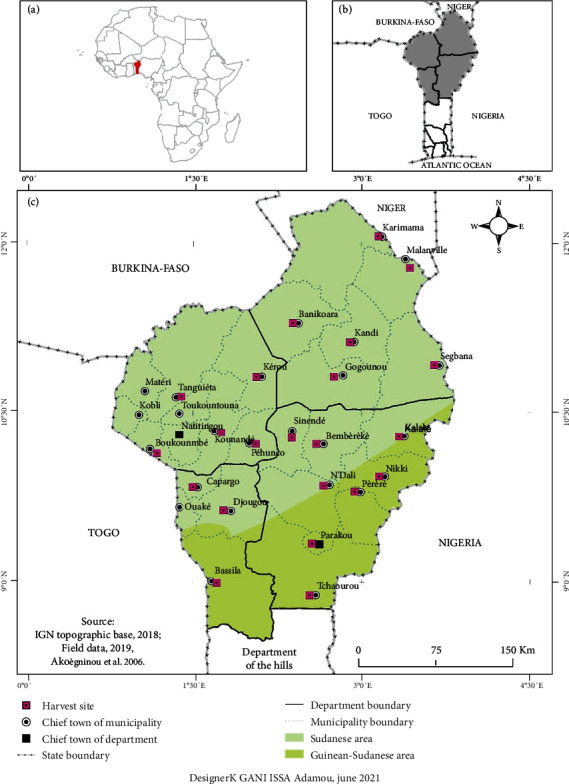
Map of Benin showing *Detarium microcarpum* seed harvesting sites.

The fruits of *Detarium microcarpum* were collected in twenty-two (22) localities in North Benin (Figure 1). They were transported to the “Research Unit in Enzymatic and Food Engineering (URGEA) of the Laboratory of Study and Research in Applied Chemistry” (LERCA) of the “Ecole Polytechnique d'Abomey-Calavi” (EPAC) of Abomey-Calavi University (UAC) for pretreatment and analysis.

### 2.2. Methods

The collected *D. microcarpum* fruits were manually peeled with a hammer to separate the seeds from the pericarp. The obtained seeds were then crushed and ground using a Moulinex type of electric grinder. The flour was wrapped in aluminium paper before being submitted to various analyses.

#### 2.2.1. Proximate Composition of *D. microcarpum* Seeds

The content of water and volatile elements, oil, total ash, total sugars (expressed as glucose), protein, and crude fiber were determined, respectively, according to the French standard NF V03-921 of the French Association for Standardization (AFNOR) [22], the Soxhlet method [23] using petroleum ether, AACC 08-01 standard [24], the phenol-sulfuric colorimetric method developed by [25], the Kjeldhal method [26], and the filter bag technique (ANKOM200) [27].

#### 2.2.2. Measurements of the Color of *D. microcarpum* Seed Meal

The color was determined according to the standards of the “International Lighting Committee” (CIE) using a Konica Minolta Chromameter in the trichromatic system (CIELAB L∗ a∗ b∗) calibrated with a white reference ceramic whose color coordinates are as follows: *X* = 0.3194, *Y* = 86.1, and *Z* = 0.3369. The recorded color parameters are L∗ (luminance index), a∗ (red saturation index), and b∗ (yellow saturation index).

#### 2.2.3. Determination of Mineral and Trace Element Content of *D. microcarpum* Seeds

The mineral elements were determined by Atomic Absorption Spectrophotometry (AAS) with a VARIANT type flame equipped with a spectra A110 software. A hollow cathode lamp and standard solutions of each mineral element were used.

#### 2.2.4. Composition of Antinutritional Factors in *D. microcarpum* Seeds

Oxalates, phytates, total phenolics, and saponins (saponosides) were determined. The method described in [28] was used to determine the oxalate content. Phytates were determined by the method of [29], and phenolics were determined by the method of Folin-Ciocalteu [30]. As for the saponin content, it was determined by the method of [31] modified by [32].

#### 2.2.5. Statistical Analysis

All determinations were performed in duplicate, and data were processed using Microsoft Excel 2010 and SPSS 25 software. An analysis of variance followed by the Student-Newman-Keuls (SNK) test was performed to compare the samples. To investigate the influence of phytogeographic zone, means were compared using the independent samples *t*-test.

## 3. Results and Discussion

### 3.1. Proximate Composition of *D. microcarpum* Seed Samples

The proximate composition of the seeds is presented in Table 1. The results show that the water and volatile element content of the collected samples varied significantly from 10.85 to 14.69%. These values are higher than 5.9% and 7.2%, reported by [16, 20] in Nigeria, respectively, but close to 10.58% and 12.5%, reported by [19, 33], respectively. This variation in moisture content in the different studies is believed to be related to environmental factors and sample drying time before analysis, as [34] showed in their study that the moisture content of a sample varies with the drying time of the sample. Furthermore, the moisture contents of *D. microcarpum* seeds obtained in different studies are lower than the 15% required as a safety limit for the storage of plant foods [35]. Therefore, *D. microcarpum* seeds can be stored for a long period of time without developing molds. Indeed, [36] showed that high moisture content decreases storage time and impacts seed quality.

As for the protein content of *D. microcarpum* seeds, it varied significantly (*P* ≤ 0.05) from 13.54 to 17.82% observed in Tanguiéta and Gogounou, respectively. These values are close to those reported in Nigeria by [16, 19, 33, 20, 21] who found 14.8%, 11.11%, 18.6%, 12.54%, and 14.22%, respectively, but lower than 35.94% reported by [18] in Nigeria. The difference between these values could be related to environmental factors, such as the difference in soil nitrogen content [37] and ecological conditions, because water deficit influences the protein content of seeds [38]. Furthermore, the values found in the present study are globally higher than the protein content (14%) of baobab (*Adansonia digitata*) flour used in Benin as a food supplement [39]. These seeds, having an interesting protein content, can constitute a protein complement in the preparation of soup and could complete the body's need for these nutrients for growth and development. Indeed, protein deficiency leads to stunted growth, muscle wasting, abnormal belly swelling, and fluid accumulation in the body [40].

As for the lipid content of *D. microcarpum* seeds, it averaged 10.05%. It varied significantly (*P* ≤ 0.05) from 8.68% to 11.90% obtained, respectively, in the samples from Boukombé and Malanville. The values of lipid content obtained from the seeds in the present study are higher than 7.42%, 7.61%, and 8.43% obtained by [20, 41, 21] in Nigeria, respectively. However, they are lower than 12%, 15%, and 14.2% as reported by [16, 17, 19] in Nigeria, respectively. These variations in seed oil content between countries and spatially could be attributed to the environmental and geological conditions of the regions where the seeds were collected [42]. Furthermore, the lipid content (8.68%–11.90%) of *D. microcarpum* seeds characterized in the present work is lower than that of industrial oilseeds, for example shea (51.86%) [43]. Thus, *D. microcarpum* seed cannot be considered an oilseed. However, seeds can be incorporated into food preparations, including the thickening of sauces, without fear of obesity and cardiovascular disease. Indeed, [44] reported that low-fat foods reduce cholesterol levels and obesity.

As for the total sugar content, it averaged 25.54% and ranged from 19.69% to 32.04%. No significant variation (*P* ≥ 0.05) was observed between the total sugar contents of the *D. microcarpum* seed samples studied. These values are lower than 42.22%, 54.4%, and 48% obtained by the difference in Nigeria by [16, 18, 19], respectively. Moreover, since these contents are relatively high, D. *microcarpum* seeds can be an energy source for the body. Indeed, total sugar is the primary energy source used by the body [45]. Furthermore, seeds can be used in the manufacture of commercial products such as glucose.

The crude fiber content of *D. microcarpum* seed samples ranged from 19.78% to 34.24% observed in Boukombé and Nikki, respectively. The analysis of variance revealed a significant variability (*P* ≤ 0.05) within the samples. These values reflect a relative richness in crude fiber compared to the crude fiber content of baobab seeds (Adansonia digitata) which is 21.2% DM as reported by [39]. On the other hand, the values in the present study are higher than 10.7%, 3.42%, and 3.5% reported in Nigeria by [16, 18, 19], respectively, but lower than 50% reported by [46]. The variations in crude fiber content observed in the present study, between countries, and in space are certainly due to the environmental conditions undergone by the seeds. Crude fiber is a compound that has the properties of providing a satisfactory dietary balance, facilitating intestinal transit, and softening stools, thus preventing constipation. They also reduce cholesterol levels and the risk of diseases such as coronary heart disease, hypertension, and cancer [47].

The average ash content of the *D. microcarpum* seeds studied was 2.47% DM. It varied significantly (*P* ≤ 0.05) from 1.5% to 3.49% observed, respectively, in Gogounou and Bassila. Our data are close to those reported in Nigeria by [16, 18, 19, 33] who found 2.2%, 2.77%, 3.2% and 3.49%, respectively. These data reflect a relative richness in mineral elements compared to those of maize, millet, and sorghum with contents of 1.50%, 1.90%, and 1.60% MS, respectively [48]. The variations in ash content of *D. microcarpum* seeds are certainly due to environmental conditions, especially the different nature of the soils.

### 3.2. Color Parameters of *D. microcarpum* Seed Samples

Table 2 shows the color parameters of *Detarium microcarpum* seeds. It shows that for an average luminance or brightness of 63.98, the red (a∗) and yellow (b∗) saturation of *D. microcarpum* seeds averaged 7.23 and 8.89, respectively. These seed color parameters varied significantly (*P* ≤ 0.05) from 60.45 to 67.64 for luminance; from 5.44 to 8.86 for red saturation; and from 8.24 to 9.28 for yellow saturation. The intensities (quantities) of the yellow hues (b∗) are slightly higher than those of the red hues (a∗), and this is true for all samples analyzed (Table 2).

### 3.3. Mineral Elements of *D. microcarpum* Seed Samples

The contents of some mineral elements could be determined and are presented in Table 3 below. It was found that potassium was the main mineral element in *D. microcarpum* seeds with an average content of 8541.85 mg/kg. It varied significantly (*P* ≤ 0.05) from 6141.88 to 12305.16 mg/kg. The highest value was observed in seeds harvested in Kerou while the lowest was observed in Péhunco. Our data are lower than those reported by [18, 21, 19] who found 23900 mg/kg, 105000 mg/kg and 35000 mg/kg, respectively, in Nigeria; but [49] in Nigeria found 6260 mg/kg which is between the values in the present study. Since *D. microcarpum* seeds are rich in potassium, then they can be used to reduce the risk of hypertension. Indeed, [50] showed that a diet rich in potassium reduces the risk of hypertension. In addition, [51] reported that potassium in intracellular and extracellular fluid in humans helps maintain electrolyte balance and membrane fluidity.

The calcium content of *D. microcarpum* seeds is 1802.27 mg/kg and ranges from 1254.47 to 2168.62 mg/kg. The analysis of variance shows us a significant difference (*P* ≤ 0.05) between the calcium contents of the different samples. The highest content was observed in Malanville while the seeds from Segbana presented the lowest content. These values are lower than those reported by [18, 21, 19] who found 2700 mg/kg, 23000 mg/kg, and 11500 mg/kg, respectively, in Nigeria; but they are higher than the 680 mg/kg reported by [49] in Nigeria. Calcium is a mineral necessary for dentition and ossification.

Regarding the magnesium content of *D. microcarpum* seeds, it varied significantly (*P* ≤ 0.05) from 1298.87 to 2533.06 mg/kg observed in Boukombé and Kouandé, respectively. These values are higher than those reported by [18, 21, 49] who found 700 mg/kg, 220 mg/kg, and 740 mg/kg, respectively, in Nigeria; but they are lower than 10500 mg/kg reported by [19] in Nigeria. Magnesium is a transmitter of nerve impulses and an activator of coenzymes in carbohydrate and protein metabolism [52].

As for the manganese content of *D. microcarpum* seeds, it is 135.54 mg/kg on average. The analysis of variance showed a significant variation (*P* ≤ 0.05) from 75.18 to 307.23 mg/kg obtained, respectively, in Banikoara and Djougou. The value 195 mg/kg reported in Nigeria by [49] is located between the values of the present study. Our values are much higher than those of [21] who found 1.7 mg/kg in Nigeria. Manganese is required for normal bone metabolism, enzymatic reactions, and maintenance of normal nerve, brain, and thyroid functions [53]. In addition, [54] reported that manganese acts as a catalyst and cofactor in many enzymatic processes such as mucopolysaccharide and glycoprotein synthesis, involved in fatty acid and cholesterol synthesis.

The mineral elements investigated in *D. microcarpum* seeds, sodium, and iron are present in low levels compared to the other elements investigated in the present study. Sodium content varied significantly (*P* ≤ 0.05) from 53.52 to 136.19 mg/kg as observed in Bembèrèkè and Banikoara, respectively. These values are lower than those reported by [18, 19, 49] who found 2200 mg/kg, 2380 mg/kg, and 450 mg/kg, respectively, in Nigeria, but higher than 28.29 mg/kg reported by [21] in Nigeria. [51] states that sodium in intracellular and extracellular fluid in humans helps to maintain electrolyte balance and membrane fluidity.

For the iron content, it varied significantly (*P* ≤ 0.05) from 28.46 to 181.42 mg/kg observed in Boukombé and Copargo, respectively. Our values are lower than those reported in Nigeria by [18, 19, 49] who found 810 mg/kg, 3120 mg/kg, and 200 mg/kg, respectively; but they are higher than 6 mg/kg reported by [21] in Nigeria. Iron is an important element for pregnant women, lactating mothers, and infants to prevent anemia [55]. The variations in the contents of the different mineral elements observed in the present study, between countries, and in space could be related to the different nature of the soils.

These values are high compared to those of maize whose calcium, potassium, magnesium, sodium, and iron contents are 145.00, 700.00, 220.00, 175.00, and 27.50 mg/kg DM, respectively [56]. The richness of *D. microcarpum* seeds in these different minerals gives it a prominent place in human and animal nutrition.

### 3.4. Antinutritional Factors of *D. microcarpum* Seed Samples

Table 4 presents the antinutritional factors sought in *D. microcarpum* oilcake. These substances are known to reduce the bioavailability of nutrients [48], particularly proteins and minerals [57]. From Table 4, it is clear that the cakes contain total phenols, phytates, oxalates, and saponins with average contents of 4.89%, 0.67%, 1.73%, and 3.08%, respectively. Except for oxalate contents, total phenols, phytates, and saponin contents varied significantly (*P* ≤ 0.05) from 3.13% to 7.61%, from 0.37% to 0.87%, and from 1.35% to 4.59%, respectively.

The oxalate (0.16%) and phytate (0.26%) contents reported in Nigeria by [33] are lower than those obtained in the present study. However, our values are lower than 5.57% obtained by [18] in Nigeria. The saponin contents of 0.13% and 0.20% obtained by [18, 33], respectively, as well as the total phenol content 0.073% obtained by [16] are lower than the values in the present study. This difference observed between the different results could be due to the environmental conditions experienced by the seeds.

Phytic acid is an antinutritional factor, known as a major inhibitor of the absorption of iron and calcium, zinc, and other minerals [58]. Phytate levels obtained in *D. microcarpum* seed meals are not negligible. However, the toxicity levels of these antinutrients have not been established [59]. However, treatments such as soaking or fermentation and cooking before consumption of these seeds would be necessary for the improvement of their quality [48, 60]. On the other hand, phytic acid can be used as an antioxidant in food products by inhibiting iron-catalyzed oxidative reactions [61].

Oxalates have also been reported to inhibit the absorption of minerals, particularly calcium, magnesium, zinc, and mercury [62]. The calcium oxalate precipitates formed are deposited in the kidneys promoting kidney stones [63]. The maximum tolerable limit of oxalate is between 0.2 and 0.5 g/100 g [64].

This implies that above this value, the adverse effects of oxalate in the human body, namely, limited bioavailability of minerals (Ca, Na, and K), kidney stones (calcium oxalate), and severe irritation of the intestinal wall, will be experienced [65]. The values obtained in the present study are much higher than the maximum tolerable limit. Cooking of *D. microcarpum* seeds before processing or consumption is essential. Indeed, the high content of oxalate in foodstuffs can be reduced if they are precooked before processing or consumption [66].

Phenolic compounds are known for their antioxidant properties [67], and they allow grain storage because they prevent losses due to premature germination and mold damage [68]. However, they are also substances that have a negative effect on the digestibility of nutrients. Indeed, the chelation property of phenolic compounds contributes to their antioxidant activity but, at the same time, to their inhibiting effect on the bioavailability of minerals [65].

High saponin content causes gastroenteritis manifested by diarrhea and dysentery [59]. However, saponins have cholesterol-lowering properties [69] and play a major role in the treatment of inflamed tissues as well as in cancer prevention [70].

Furthermore, the phenolic compound contents of *D. microcarpum* seeds obtained in the present study are between 0.2 and 10.3% DM reported by [71] on Sorghum grains.

### 3.5. Influence of Phytogeographic Zones on the Physicochemical Characteristics of *Detarium microcarpum* Seed Samples

The physicochemical characteristics of *D. microcarpum* seeds, evaluated in the present study, were all subjected to the independent samples *t*-test to estimate the influence of phytogeographic zones on the means of the said characteristics. The results obtained are presented in Table 5 below. It is shown that except the total sugar content, there is no significant difference (*P* ≥ 0.05) between the means of physicochemical characteristics of seeds from the two phytogeographic zones. Thus, on average, the physicochemical characteristics of the Sudanian and Sudano-Guinean zones are close except the total sugar content. In other words, the phytogeographic zone does not influence the physicochemical characteristics of *D. microcarpum* seeds except total sugars.

## 4. Conclusion

The present study provided scientific data on the proximate composition and antinutritional factors of *Detarium microcarpum* seeds from Benin. According to the results obtained, *Detarium microcarpum* seeds can be good sources of total sugars, crude fiber, protein, lipid, and minerals such as potassium, calcium, magnesium, and manganese. The obtained oxalate and phytate contents can reduce the bioavailability of nutrients, especially minerals contained in the seeds. On average, the physicochemical characteristics of the Sudanian and Sudano-Guinean zones are close except the total sugar content.

## Figures and Tables

**Table 1:** Proximate composition of *Detarium microcarpum* seeds in North Benin.

Areas	Municipalities	Water and volatile matter content (%)	Protein (% DM)	Total sugar content (% DM)	Lipid content (% DM)	Raw fibers (% DM)	Ash content (% DM)
Sudanian	Banikoara	13.40 ± 0.20^de^	15.66 ± 0.25^b^	24.43 ± 0.65^a^	10.60 ± 0.12^c^	30.93 ± 1.75^bc^	2.52 ± 0.18^abc^
	Kandi	12.40 ± 0.43^bc^	15.57 ± 0.12^b^	26.8 ± 3.08^a^	10.15 ± 0.19^bc^	28.68 ± 3.07^bc^	2.18 ± 1.17^abc^
	Malanville	12.24 ± 0.19^bc^	15.05 ± 0.62^ab^	22.83 ± 1.23^a^	11.90 ± 0.74^d^	31.79 ± 0.87^bc^	2.08 ± 1.03^abc^
	Karimama	13.01 ± 0.06^cd^	15.14 ± 0.27^ab^	21.925 ± 1.59^a^	9.01 ± 0.08^ab^	26.49 ± 0.64^bc^	2.57 ± 0.04^abc^
	Ségbana	10.85 ± 0.04^a^	15.44 ± 0.10^ab^	27.73 ± 2.33^a^	10.33 ± 0.07^c^	31.29 ± 0.46^bc^	2.73 ± 0.25^abc^
	Gogounou	12.51 ± 0.14^bcd^	17.82 ± 0.21^b^	22.12 ± 1.67^a^	8.76 ± 0.29^a^	29.32 ± 2.93^bc^	1.50 ± 0.01^a^
	Kérou	12.31 ± 0.29^bc^	15.43 ± 0.52^ab^	21.62 ± 2.21^a^	10.24 ± 0.23^bc^	31.14 ± 0.18^bc^	2.95 ± 0.08^bc^
	Kouandé	12.65 ± 0.22^bcd^	14.42 ± 0.02^ab^	19.69 ± 2.92^a^	9.79 ± 0.23^abc^	31.54 ± 4.85^bc^	2.35 ± 0.07^abc^
	Péhunco	13.46 ± 0.54^de^	14.03 ± 0.01^ab^	23.98 ± 2.31^a^	9.85 ± 0.32^abc^	27.86 ± 4.65^bc^	2.35 ± 0.35^abc^
	Tanguiéta	12.03 ± 0.26^bc^	13.54 ± 0.44^a^	25.17 ± 1.63^a^	9.82 ± 0.28^abc^	31.51 ± 1.61^bc^	2.57 ± 0.10^abc^
	Boukombé	14.69 ± 0.42^f^	15.41 ± 0.83^ab^	26.86 ± 1.05^a^	8.68 ± 0.02^a^	19.78 ± 1.13^a^	2.92 ± 0.04^abc^
	Bembèrèkè	11.78 ± 0.15^b^	15.29 ± 0.33^ab^	23.48 ± 4.86^a^	10.12 ± 0.10^bc^	31.99 ± 0.98^bc^	2.27 ± 0.18^abc^
	Sinendé	12.75 ± 0.50^bcd^	15.72 ± 0.34^b^	26.65 ± 2.38^a^	9.65 ± 0.01^abc^	32.22 ± 1.17^bc^	2.32 ± 0.18^abc^
	Djougou	13.85 ± 0.14^e^	14.86 ± 0.92^ab^	23.82 ± 5.78^a^	10.40 ± 0.07^c^	30.04 ± 1.31^bc^	3.17 ± 0.11^bc^
	Copargo	12.63 ± 0.06^bcd^	14.42 ± 0.92^ab^	24.95 ± 0.95^a^	10.17 ± 0.10^bc^	28.97 ± 4.58^bc^	1.76 ± 0.01^ab^
Sudanese-Guinean	N'Dali	12.68 ± 0.19^bcd^	15.08 ± 0.22^ab^	30.41 ± 5.59^a^	10.44 ± 0.21^c^	30.62 ± 1.22^bc^	2.33 ± 0.04^abc^
	Parakou	11.83 ± 0.42^b^	14.87 ± 0.93^ab^	27.14 ± 2.69^a^	9.43 ± 0.08^abc^	27.31 ± 2.42^bc^	2.35 ± 0.14^abc^
	Pèrèrè	12.31 ± 0.40^bc^	14.66 ± 0.04^ab^	27.96 ± 4.16^a^	10.12 ± 0.41^bc^	32.71 ± 0.54^bc^	2.85 ± 0.15^abc^
	Kalalé	12.85 ± 0.16^bcd^	14.22 ± 0.61^ab^	25.68 ± 4.55^a^	10.22 ± 0.78^bc^	32.59 ± 3.22^bc^	2.17 ± 0.11^abc^
	Tchaourou	12.80 ± 0.25^bcd^	15.68 ± 0.04^b^	32.04 ± 2.96^a^	9.75 ± 0.30^abc^	32.03 ± 4.33^bc^	2.58 ± 0.04^abc^
	Nikki	12.14 ± 0.21^bc^	15.07 ± 0.59^ab^	27.41 ± 3.59^a^	11.63 ± 0.69^d^	34.24 ± 0.59^c^	2.32 ± 0.24^abc^
	Bassila	13.83 ± 0.04^e^	15.63 ± 0.37^b^	29.32 ± 2.96^a^	10.15 ± 0.52^bc^	22.785 ± 2.29^ab^	3.49 ± 0.08^c^
	Total	12.68 ± 0.84	15.14 ± 0.90	25.54 ± 3.75	10.05 ± 0.79	29.81 ± 3.79	2.47 ± 0.51

Values with different letters in the same column are significantly different at the 5% level.

**Table 2:** Color parameters of *Detarium microcarpum* seeds.

Areas	Municipalities	l∗	a∗	b∗
Sudanian	Banikoara	64.38 ± 0.01^h^	6.74 ± 0.06^c^	8.71 ± 0.06^bc^
	Kandi	62.88 ± 0.26^d^	7.58 ± 0.03^f^	9.28 ± 0.01^f^
	Malanville	63.43 ± 0.01^e^	7.52 ± 0.05^f^	8.82 ± 0.04^cd^
	Karimama	62.54 ± 0.12^c^	8.25 ± 0.06^i^	8.89 ± 0.06^de^
	Ségbana	64.31 ± 0.02^h^	7.55 ± 0.04^f^	9.00 ± 0.03^e^
	Gogounou	60.45 ± 0.04^a^	8.86 ± 0.07^j^	9.15 ± 0.06^f^
	Kérou	62.61 ± 0.01^c^	7.73 ± 0.05^g^	9.18 ± 0.07^f^
	Kouandé	61.95 ± 0.00^b^	7.44 ± 0.03^f^	8.89 ± 0.03^de^
	Péhunco	62.64 ± 0.01^c^	7.44 ± 0.08^f^	8.89 ± 0.01^de^
	Tanguiéta	62.68 ± 0.02^c^	7.73 ± 0.09^g^	8.24 ± 0.00^a^
	Boukombé	65.77 ± 0.00^k^	6.05 ± 0.04^b^	8.64 ± 0.03^b^
	Bembèrèkè	62.46 ± 0.01^c^	7.91 ± 0.07^h^	9.25 ± 0.06^f^
	Sinendé	67.64 ± 0.03^m^	6.79 ± 0.02^c^	8.97 ± 0.04^e^
	Djougou	64.02 ± 0.13^g^	6.67 ± 0.08^c^	8.29 ± 0.03^a^
	Copargo	65.57 ± 0.04^j^	6.96 ± 0.02^d^	8.78 ± 0.03^cd^
Sudanese-Guinean	N'Dali	63.80 ± 0.01^f^	6.94 ± 0.01^d^	8.77 ± 0.02^cd^
	Parakou	64.41 ± 0.13^h^	7.16 ± 0.01^e^	8.70 ± 0.04^bc^
	Pèrèrè	65.86 ± 0.09^k^	6.60 ± 0.01^c^	8.72 ± 0.03^bc^
	Kalalé	64.38 ± 0.04^h^	7.14 ± 0.01^e^	8.61 ± 0.03^b^
	Tchaourou	64.53 ± 0.12^h^	7.58 ± 0.03^f^	9.28 ± 0.01^f^
	Nikki	64.80 ± 0.00^i^	7.10 ± 0.06^e^	9.22 ± 0.06^f^
	Bassila	66.49 ± 0.05^l^	5.44 ± 0.04^a^	9.27 ± 0.05^f^
	Total	63.98 ± 1.65	7.23 ± 0.72	8.89 ± 0.30

Values with different letters in the same column are significantly different at the 5% level.

**Table 3:** Contents of some mineral elements in *Detarium microcarpum* seeds.

Areas	Municipalities	Sodium (Na), mg/kg	Potassium (K), mg/kg	Manganese (Mn), mg/kg	Calcium (Ca), mg/kg	Magnesium (mg), mg/kg	Iron (Fe), mg/kg
Sudanian	Banikoara	136.19 ± 0.01^v^	7117.31 ± 0.07^e^	75.18 ± 0.01^b^	1590.55 ± 0.01^e^	1708.35 ± 0.02^l^	57.54 ± 0.04^q^
	Kandi	64.56 ± 0.01^f^	8420.44 ± 0.04^n^	78.94 ± 0.01^c^	1254.48 ± 0.01^a^	1577.07 ± 0.01^h^	48.87 ± 0.04^j^
	Malanville	76.67 ± 0.04^l^	6913.85 ± 0.06^d^	82.08 ± 0.02^g^	2168.62 ± 0.02^u^	1602.65 ± 0.03^j^	88.75 ± 0.03^u^
	Karimama	56.94 ± 0.04^b^	8146.73 ± 0.03^l^	153.26 ± 0.04^s^	1274.47 ± 0.05^b^	1924.62 ± 0.01^q^	37.17 ± 0.01^e^
	Ségbana	66.94 ± 0.04^h^	8446.73 ± 0.03^p^	133.26 ± 0.04^n^	1254.47 ± 0.05^a^	1524.62 ± 0.01^g^	47.17 ± 0.01^h^
	Gogounou	70.42 ± 0.01^j^	8100.84 ± 0.03^k^	79.79 ± 0.01^d^	1890.04 ± 0.03^p^	1672.48 ± 0.03^k^	54.51 ± 0.01^m^
	Kérou	66.23 ± 0.01^g^	12505.46 ± 0.07^v^	81.64 ± 0.01^f^	1963.67 ± 0.01^q^	1745.16 ± 0.01^o^	36.63 ± 0.01^d^
	Kouandé	61.83 ± 0.01^d^	12305.16 ± 0.01^u^	113.85 ± 0.03^j^	1650.63 ± 0.01^j^	2533.06 ± 0.05^v^	55.33 ± 0.04^o^
	Péhunco	83.85 ± 0.05^o^	6141.88 ± 0.02^a^	142.57 ± 0.03^r^	1750.65 ± 0.04^l^	1444.15 ± 0.06^e^	88.53 ± 0.01^t^
	Tanguiéta	98.42 ± 0.01^s^	7193.23 ± 0.04^f^	100.43 ± 0.01^i^	1748.56 ± 0.04^k^	1722.96 ± 0.03^m^	81.07 ± 0.01^s^
	Boukombé	83.07 ± 0.01^n^	8408.14 ± 0.04^m^	195.07 ± 0.04^u^	1606.83 ± 0.03^h^	1298.87 ± 0.03^a^	28.46 ± 0.01^a^
	Bembèrèkè	53.52 ± 0.03^a^	7214.06 ± 0.04^g^	94.69 ± 0.01^h^	1759.06 ± 0.04^m^	1498.87 ± 0.01^f^	36.12 ± 0.03^c^
	Sinendé	80.27 ± 0.03^m^	10110.66 ± 0.02^r^	131.04 ± 0.01^m^	1800.17 ± 0.01^n^	1982.27 ± 0.03^r^	55.19 ± 0.01^n^
	Djougou	69.52 ± 0.01^i^	6699.53 ± 0.02^c^	307.23 ± 0.01^v^	1983.29 ± 0.01^r^	1358.64 ± 0.02^b^	53.23 ± 0.04^l^
	Copargo	62.34 ± 0.03^e^	7783.16 ± 0.01^h^	138.74 ± 0.01^q^	2024.23 ± 0.03^s^	1365.25 ± 0.01^c^	181.42 ± 0.01^v^
Sudanese-Guinean	N'Dali	57.12 ± 0.01^c^	7993.36 ± 0.02^i^	81.44 ± 0.01^e^	2099.92 ± 0.03^t^	1369.28 ± 0.02^d^	61.44 ± 0.01^r^
	Parakou	84.16 ± 0.05^p^	10115.96 ± 0.02^s^	135.54 ± 0.01^o^	1802.27 ± 0.01^o^	1987.27 ± 0.03^s^	50.16 ± 0.04^k^
	Pèrèrè	88.94 ± 0.01^r^	6408.83 ± 0.01^b^	161.26 ± 0.02^t^	1649.82 ± 0.01^i^	1780.64 ± 0.04^p^	56.06 ± 0.02^p^
	Kalalé	84.98 ± 0.04^q^	8443.15 ± 0.06^o^	137.54 ± 0.01^p^	1477.60 ± 0.01^d^	1592.64 ± 0.04^h^	39.72 ± 0.01^f^
	Tchaourou	117.85 ± 0.01^u^	11684.26 ± 0.01^t^	122.24 ± 0.01^l^	1605.83 ± 0.03^g^	2030.35 ± 0.01^t^	40.54 ± 0.03^g^
	Nikki	112.47 ± 0.03^t^	9768.63 ± 0.03^q^	64.23 ± 0.04^a^	1593.46 ± 0.02^f^	1741.86 ± 0.01^n^	47.84 ± 0.01^i^
	Bassila	72.75 ± 0.00^k^	7999.47 ± 0.04^j^	119.98 ± 0.04^k^	1354.58 ± 0.01^c^	2266.51 ± 0.01^u^	33.64 ± 0.04^b^
	Total	79.50 ± 20.89	8541.85 ± 1800.90	124.09 ± 52.26	1695.60 ± 263.40	1714.89 ± 306.15	58.15 ± 31.66

Values with different letters in the same column are significantly different at the 5% level.

**Table 4:** Antinutritional factors of *Detarium microcarpum* cake.

Areas	Municipalities	Oxalate (%)	Phytate (%)	Total phenols (%)	Saponin (%)
Sudanian	Banikoara	1.68 ± 0.48^a^	0.37 ± 0.03^a^	4.93 ± 0.11^bcde^	4.59 ± 0.12^e^
	Kandi	1.67 ± 0.47^a^	0.87 ± 0.06^e^	6.62 ± 0.09^f^	2.41 ± 0.13^ab^
	Malanville	1.67 ± 0.47^a^	0.76 ± 0.13^cde^	4.70 ± 0.03^abcde^	1.35 ± 0.21^a^
	Karimama	1.34 ± 0.00^a^	0.76 ± 0.05^cde^	5.78 ± 0.66^def^	3.55 ± 0.83^cde^
	Ségbana	1.01 ± 0.47^a^	0.51 ± 0.07^abc^	5.01 ± 0.04^cde^	4.01 ± 0.08^cde^
	Gogounou	2.03 ± 0.01^a^	0.60 ± 0.21^abcde^	7.61 ± 0.54^g^	3.53 ± 0.04^cde^
	Kérou	1.34 ± 0.95^a^	0.79 ± 0.04^cde^	3.43 ± 0.02^abc^	1.44 ± 0.37^a^
	Kouandé	2.03 ± 0.95^a^	0.64 ± 0.01^bcde^	5.04 ± 0.20^de^	1.81 ± 0.29^a^
	Péhunco	1.69 ± 0.47^a^	0.83 ± 0.01^de^	3.38 ± 0.02^ab^	3.48 ± 0.74^cde^
	Tanguiéta	1.34 ± 0.95^a^	0.72 ± 0.06^bcde^	5.77 ± 0.35^def^	4.11 ± 0.02^de^
	Boukombé	1.69 ± 0.47^a^	0.57 ± 0.06^abcd^	3.13 ± 0.11^a^	2.91 ± 0.06^bc^
	Bembèrèkè	1.68 ± 0.47^a^	0.53 ± 0.01^abc^	5.73 ± 0.37^def^	4.47 ± 0.47^e^
	Sinendé	1.69 ± 0.47^a^	0.60 ± 1.11^abcde^	5.00 ± 0.28^cde^	3.11 ± 0.01^bcd^
	Djougou	2.36 ± 0.47^a^	0.76 ± 0.05^cde^	3.33 ± 0.09^a^	2.40 ± 0.00^ab^
	Copargo	1.69 ± 0.48^a^	0.70 ± 0.04^bcde^	4.52 ± 0.17^abcde^	3.75 ± 0.35^cde^
Sudanese-Guinean	N'Dali	2.02 ± 0.95^a^	0.73 ± 0.08^cde^	4.11 ± 0.59^abcd^	3.70 ± 0.42^cde^
	Parakou	2.01 ± 0.00^a^	0.72 ± 0.06^bcde^	4.65 ± 0.49^abcde^	2.29 ± 0.12^ab^
	Pèrèrè	1.68 ± 0.48^a^	0.77 ± 0.10^cde^	4.13 ± 0.11^abcd^	4.05 ± 0.07^de^
	Kalalé	2.02 ± 0.00^a^	0.82 ± 0.04^de^	5.77 ± 0.13^def^	4.14 ± 0.04^de^
	Tchaourou	1.35 ± 0.95^a^	0.58 ± 0.04^abcd^	4.52 ± 1.10^abcde^	2.40 ± 0.01^ab^
	Nikki	1.68 ± 0.47^a^	0.78 ± 0.11^cde^	5.99 ± 0.44^ef^	2.92 ± 0.07^bc^
	Bassila	2.35 ± 0.48^a^	0.46 ± 0.00^ab^	4.52 ± 0.00^abcde^	1.45 ± 0.36^a^
	Total	1.73 ± 0.49	0.67 ± 0.14	4.89 ± 1.15	3.08 ± 1.03

Values with different letters in the same column are significantly different at the 5% level.

**Table 5:** Influence of phytogeographical zones on the physicochemical characteristics of *Detarium microcarpum* seeds.

		Sudanian	Sudanese-Guinean
Proximate composition	Moisture	12.70 ± 0.91^a^	12.63 ± 0.64^a^
	Protein	15.19 ± 0.97^a^	15.03 ± 0.52^a^
	Total sugar	21.09 ± 2.01^a^	24.76 ± 2.01^b^
	Lipid content	9.96 ± 0.79^a^	10.25 ± 0.69^a^
	Raw fibers	27.80 ± 4.88^a^	28.96 ± 7.77^a^
	Ash content	2.41 ± 0.44^a^	2.58 ± 0.46^a^
Color settings	L∗	63.55 ± 1.78^a^	64.89 ± 0.94^a^
	a∗	7.41 ± 0.70^a^	6.85 ± 0.69^a^
	b∗	8.86 ± 0.31^a^	8.94 ± 0.30^a^
Mineral components	Na	75.38 ± 20.51^a^	88.32 ± 21.21^a^
	K	8367.14 ± 1894.72^a^	8916.23 ± 1733.54^a^
	Mn	127.18 ± 60.63^a^	117.46 ± 33.69^a^
	Ca	1714.65 ± 283.73^a^	1654.78 ± 240.69^a^
	Mg	1663.93 ± 311.53^a^	1824.08 ± 298.32^a^
	Fe	63.33 ± 37.52^a^	47.05 ± 9.78^a^
Antinutrients	Oxalate	1.66 ± 0.33^a^	1.87 ± 0.33^a^
	Phytate	0.67 ± 0.14^a^	0.69 ± 0.13^a^
	Total phenols	4.93 ± 1.28^a^	4.81 ± 0.76^a^
	Saponin	3.13 ± 1.05^a^	2.99 ± 1.02^a^

Values with different letters in the same line are significantly different at the 5% level.

## Data Availability

There is no data to support the findings of this study.
